# Genome-Wide Identification and Characterization of the NAC Transcription Factor Family in *Sinojackia xylocarpa* Hu

**DOI:** 10.3390/plants15081163

**Published:** 2026-04-09

**Authors:** Yifei Hong, Yaoyuan Wang, Yifan Duan, Sheng Zhu

**Affiliations:** College of Life Sciences, Nanjing Forestry University, Nanjing 210037, China; hyf000609@163.com (Y.H.); wangyy200111@163.com (Y.W.)

**Keywords:** expression patterns, gene duplication, NAC transcription factor, *Sinojackia xylocarpa*

## Abstract

NAC (NAM, ATAF1/2 and CUC2) transcription factors constitute one of the largest plant-specific transcription factor families and play pivotal roles in plant growth, development, and responses to environmental stresses. Systematic characterization of *NAC* genes is essential for understanding regulatory networks underlying key agronomic and adaptive traits. As a conservation-priority woody species with distinctive biological and horticultural value, *Sinojackia xylocarpa* Hu lacks comprehensive knowledge of its NAC repertoire, and elucidating its NAC family will facilitate functional studies related to development and environmental adaptation. Based on whole-genome data of *S. xylocarpa*, we conducted a systematic survey and characterization of the NAC transcription factor family. In total, 115 *SxyNAC* genes encoding the conserved NAC domain were identified, and their loci were unevenly distributed across 12 chromosomes. Analyses of gene-duplication modes and collinearity indicated that whole-genome/segmental duplication events were the major driving force for the expansion of this family. Phylogenetic relationships, gene structures, and conserved motifs classified the *SxyNAC* members into 15 subfamilies, revealing a highly conserved N-terminal NAC domain and a markedly diversified C-terminal regulatory region with pronounced member- and lineage-specific differences. Promoter cis-element prediction showed extensive enrichment of light-responsive, phytohormone-responsive, and stress-related elements, suggesting that *SxyNAC* genes may participate in coordinated regulation of multiple environmental cues and endogenous hormone pathways. Transcriptome data from six fruit developmental stages, together with qRT-PCR validation of ten representative genes, demonstrated diverse temporal and tissue-specific expression patterns during fruit development and close associations with fruit growth regulation. Overall, our findings establish a framework for exploring the evolutionary trajectories and functional diversification of *NAC* genes in *S. xylocarpa*, and they offer a valuable resource for NAC-family research and conservation-focused functional genomics in other rare or threatened plant species.

## 1. Introduction

NAC transcription factors (originally designated as NAM in Petunia, ATAF1/2 and CUC2 in *Arabidopsis thaliana*) constitute one of the largest and most plant-specific families of transcriptional regulators, recognized as central components of gene regulatory networks in plants. Structurally, NAC proteins are defined by two principal domains, namely a conserved N-terminal NAC domain and a highly variable C-terminal transcriptional regulatory domain (TRD). The N-terminal NAC domain, consisting of ~150 amino acids, acts as a DNA-binding domain (DBD) that recognizes *cis*-acting elements within the promoters of target genes, thereby modulating transcriptional activity [1]. Owing to this modular configuration, NAC transcription factors integrate diverse biological processes, including plant growth and development, secondary metabolism, and environmental adaptation, serving as a critical conduit between external signaling and metabolic regulation [2].

Evidence accumulated over the past few years suggests that *NAC* family genes are tightly linked to the buildup of specialized metabolites, underscoring the functional versatility of this group in governing metabolic circuitry. In the model plant *A. thaliana*, *ANAC078* activates *CHS*, *F3H*, and *DFR* genes under high-light stress, leading to enhanced anthocyanin biosynthesis and improved photoprotection [3]. Another member, *ANAC042*/*JUB1*, is induced upon powdery mildew infection and regulates phenylpropanoid biosynthetic genes through a salicylic acid (SA)-independent pathway, illustrating a defense-related role for NAC proteins in metabolic reprogramming [4]. Similarly, in *Vitis vinifera*, the defense-associated gene *VvNAC042_5* is strongly induced by powdery mildew colonization and promotes the transcription of *stilbene synthase* and other antimicrobial secondary metabolism genes, suggesting a conserved defense mechanism mediated by NAC factors [5].

In fruit crops, NAC transcription factors play essential roles in fruit ripening and pigment biosynthesis. In red-fleshed apple (*Malus domestica*), the abscisic acid (ABA)-induced *MdNAC1* interacts with a bZIP-type transcription factor (*MdbZIP23*) to directly bind and activate the promoters of *MdMYB10* and *MdUFGT*, thereby promoting anthocyanin accumulation [6]. This discovery revealed a novel cooperative ABA–NAC–bZIP regulatory module that controls fruit pigmentation. Similarly, *FaRIF* in *Fragaria* × *ananassa* and *NOR* in *Solanum lycopersicum* have been identified as master regulators of fruit ripening and phenylpropanoid metabolism, influencing cell wall degradation, ABA biosynthesis, and flavor formation [7,8,9].

In medicinal plants, the regulatory roles of NAC transcription factors in secondary metabolism are particularly prominent. In *Scutellaria baicalensis*, *SbNAC6* expression shows a strong positive correlation with *PAL2*, suggesting its involvement in the biosynthesis of flavonoids such as baicalin and baicalein [10]. In *Sophora tonkinensis*, genome-wide identification revealed numerous *StNAC* genes significantly correlated with the accumulation of flavonoids and alkaloids. Promoter analysis indicated enrichment of ABA- and JA-responsive elements, implying that these genes participate in both stress adaptation and secondary metabolite regulation [11]. Moreover, *CrNAC* genes in *Catharanthus roseus* were shown to participate in the biosynthesis of terpenoid indole alkaloids (TIAs), including vinblastine and vindoline, providing further evidence for the involvement of NAC factors in specialized metabolite pathways of medicinal plants [12].

In cereal crops, NAC transcription factors are closely linked to metabolic remodeling and defense. In *Oryza sativa*, *OsNAC2* interacts with the gibberellin (GA) signaling pathway to modulate plant height and lignin accumulation [13], while in *Triticum aestivum*, *TaNAC032* activates the expression of lignin biosynthetic genes, leading to reinforced cell wall deposition and improved resistance to Fusarium head blight [14]. Taken together, available evidence supports the argument that NAC transcription factors function in both hormone- and defense-associated signaling pathways, while also exerting regulatory control over lignin biosynthesis and phenylpropanoid metabolism.

*Sinojackia xylocarpa* Hu is a rare woody plant species endemic to China and is listed as a national Class II protected species (http://www.iplant.cn/bhzw/, accessed on 23 April 2025). Due to its rarity, previous studies have primarily focused on ecological conservation, seed dormancy, and phylogenetic evolution, whereas molecular-level research remains relatively limited [15,16,17]. To address this gap, we aimed to systematically analyze the NAC transcription factor family in *S. xylocarpa*, identify its members, and elucidate their structural and evolutionary characteristics. By integrating transcriptomic data from six fruit developmental stages, we further investigated the expression patterns of these genes and screened potential key candidates, offering an analytical framework to clarify the evolutionary characteristics and probable physiological roles within the NAC family of genes from *S. xylocarpa*.

In this study, we detected NAC transcription factors in the *S. xylocarpa* genome [18] and conducted comprehensive bioinformatic analyses of their family composition and evolutionary expansion. These analyses encompassed physicochemical property prediction, chromosomal localization, phylogenetic classification, and intra- and inter-specific collinearity analyses. In addition, we selected ten *NAC* genes from *S. xylocarpa* fruits and analyzed their expression profiles across six developmental time points using qRT-PCR. This analysis facilitated the identification of potential functional genes closely associated with fruit development based on temporal expression dynamics. Collectively, this work establishes fundamental genomic resources and an integrated analytical framework for the NAC family in *S. xylocarpa*, linking evolutionary features with stage-resolved fruit expression patterns to prioritize candidate regulators potentially involved in fruit enlargement, ripening, and phytohormone- and environment-responsive pathways. These results provide a solid foundation for subsequent functional verification and offer actionable molecular targets and theoretical support for the breeding, utilization, and conservation-oriented functional improvement of this threatened woody species.

## 2. Results

### 2.1. Identification and Nomenclature of NAC Genes in Sinojackia xylocarpa Hu

We detected 115 genes encoding NAC domain-containing proteins (PFAM no. PF02365) from the *S. xylocarpa* genome. These transcripts correspond one-to-one to 115 gene loci in the *S. xylocarpa* genome. In the subsequent text, all *NAC* genes are referred to using the unified custom nomenclature established in this study (e.g., *SxyNAC012*). To remain consistent with the genome annotation, the figures and tables use the original gene identifiers assigned in the annotation (e.g., Ssp09G021640). The correspondence between the original IDs and the custom IDs is provided in Table S1. Linkage group associations were not determined for genes *SxyNAC033*, *SxyNAC034*, *SxyNAC083*, *SxyNAC084*, *SxyNAC107*, and *SxyNAC111*.

The predicted SxyNAC proteins range from 154 (SxyNAC080) to 802 aa (SxyNAC057) in length, with a mean of 353.23 aa. In line with this, the estimated protein mass extends from 18,196.83 Da for SxyNAC080 to 88,831.52 Da for SxyNAC057. To comprehensively assess the physicochemical attributes of the SxyNAC family, we performed a thorough characterization of their basic properties and computed the isoelectric point (pI), GC content, aliphatic index, instability index and grand average of hydropathicity (GRAVY) for each protein. These physicochemical descriptors provide important clues for understanding the functional attributes and potential biological roles of NAC family proteins. The predicted pI values of the SxyNAC proteins range from 4.13 (SxyNAC012) to 9.72 (SxyNAC086). Across the coding regions of these *NAC* genes, the mean GC proportion is 45.47%, ranging from 36.56% for *SxyNAC080* to 56.16% for *SxyNAC041*. The aliphatic index of the SxyNAC proteins lies between 48.082 and 84.817 (normalized), with a mean value of 64.739. The relatively high aliphatic indices for most members indicate an enrichment of aliphatic amino acids (e.g., Ala, Val, Ile and Leu), a feature that is typically associated with improved thermal stability of proteins [19]. Accordingly, we infer that NAC proteins may retain relatively high stability under temperature fluctuations or various stress conditions, which is likely important for the proper execution of their functions in plant cells. The instability index of NAC family proteins is generally above 40, ranging from 28.349 to 61.385, with an average value of 45.629, suggesting that most proteins are predicted to be relatively unstable and tend to be labile under in vitro conditions. The GRAVY scores of SxyNAC proteins are all negative, from −1.159 to −0.260, with an average of −0.702. This strongly indicates that the proteins are overall hydrophilic. Negative GRAVY values are commonly associated with intracellular, particularly nuclear, localization and function, consistent with the role of NAC proteins as transcription factors. This hydrophilic property could promote cellular interactions between NAC proteins and nucleic acids (DNA/RNA) or partner proteins, which may support their roles in controlling plant development and mediating responses to both abiotic and biotic challenges. Details are summarized in Table S2.

### 2.2. Chromosomal Distribution and Synteny Analysis of NAC Genes in Sinojackia xylocarpa

**Figure 1:**
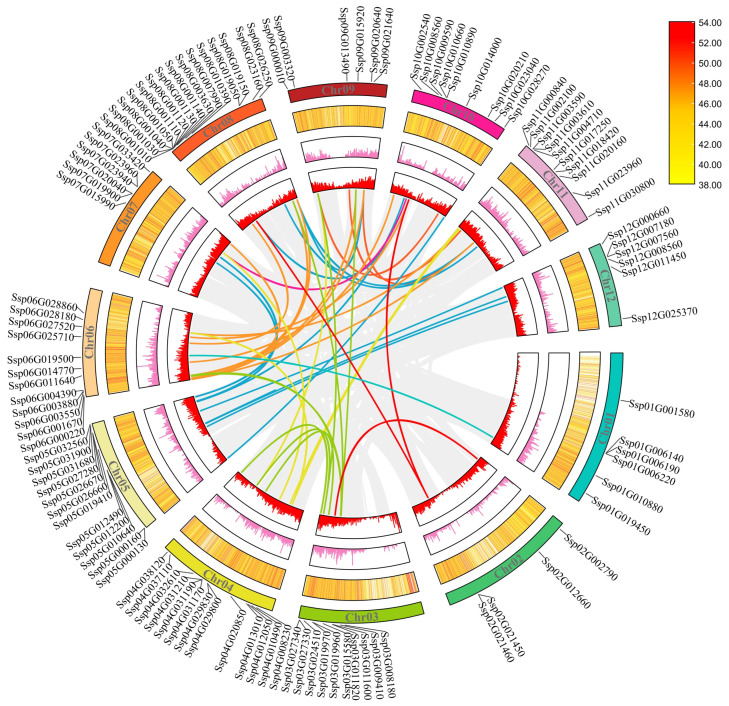
Circos plot illustrating chromosomal locations and syntenic links among *NAC* genes in *S. xylocarpa*. The outermost circle represents the 12 chromosomes (Chr01–Chr12) with the physical positions and IDs of *SxyNAC* genes. From the outside to the inside, the three tracks display GC content (heatmap), repeat sequence density and gene density along each chromosome, respectively. Coloured ribbons in the centre connect segmentally duplicated *SxyNAC* gene pairs, whereas grey ribbons indicate background collinear gene pairs across the genome. Gene labels in the figure correspond to the original genome annotation IDs; the mapping to the custom IDs is provided in Table S1.

Among the 115 *SxyNAC* genes identified, 109 are physically anchored to the 12 chromosomes of *S. xylocarpa*, with an evidently uneven distribution across pseudochromosomes (Figure 1). Table S3 summarizes the chromosomal assignments of all *SxyNAC* genes, together with their genomic coordinates (start/end positions) and strand orientation. Across chromosomes, *SxyNAC* copy numbers vary from 4 on Chr02 to 15 on Chr08. Five NAC-enriched clusters were identified on Chr04, Chr05, Chr06, Chr08, and Chr11 (Table S4).

Based on the classification of duplication modes, 63 of the 115 *SxyNAC* genes (54.8%) were assigned to the WGD/segmental duplication category, 29 (25.2%) to dispersed duplication, 13 (11.3%) to tandem duplication and 10 (8.7%) to proximal duplication. Detailed information is provided in Table S5. These results indicate that ancient whole-genome or large-segment duplication events have been the predominant driving force underlying the expansion of the *SxyNAC* gene family. Dispersed duplication, typically associated with transposition or chromosomal rearrangements, represents the second most important source, suggesting a prominent role of transposon-mediated long-distance duplication in reshaping the genomic distribution of NAC members. In contrast, tandem and proximal duplications mainly contributed to local copy amplification within restricted chromosomal regions [20].

In total, 46 collinear homologous *SxyNAC* gene pairs were identified in the chromosomal synteny analysis (Figure 1 and Figure S1). Detailed information is provided in Table S6. All duplicated gene pairs exhibit *Ka*/*Ks* ratios < 1, suggesting that post-expansion evolution of the NAC family has been governed largely by purifying selection, thereby preserving the ancestral functions of the encoded proteins [21]. The estimated divergence times of these homologous *NAC* pairs range from 1.221 to 252.546 Mya, in which T is estimated as *Ks*/(2λ), with λ = 6.5 × 10^−9^ substitutions/site/year [22,23], with detailed *Ka* and *Ks* values, *Ka*/*Ks* ratios, and divergence time estimates summarized in Table S7, spanning a broad evolutionary window and implying that the expansion of the *SxyNAC* family was not triggered by a single event but rather occurred in multiple phases. A subset of gene pairs shows extremely low *Ks* values, corresponding to divergence times of only a few million years, and may have arisen from recent tandem or segmental duplication events after species divergence. At the family level, most duplication events are concentrated within the 30–100 Mya interval, with approximately 60% of the duplicated pairs falling into this range, suggesting that this period represents a relatively prominent expansion peak for the *SxyNAC* family. Only a few gene pairs have divergence times <30 Mya, indicating that a limited number of recent duplication events continued to occur after the split of closely related lineages; meanwhile, roughly one-third of the duplicated pairs diverged >100 Mya, reflecting the long-term retention of ancient copies during evolution. Collectively, these findings demonstrate that the expansion of the *SxyNAC* gene family in *S. xylocarpa* comprises both ancient duplications preserved from early evolutionary stages and more recent events that occurred during later species evolution, providing time-scale evidence for the generation of member diversity in this family [24,25].

**Figure 2:**
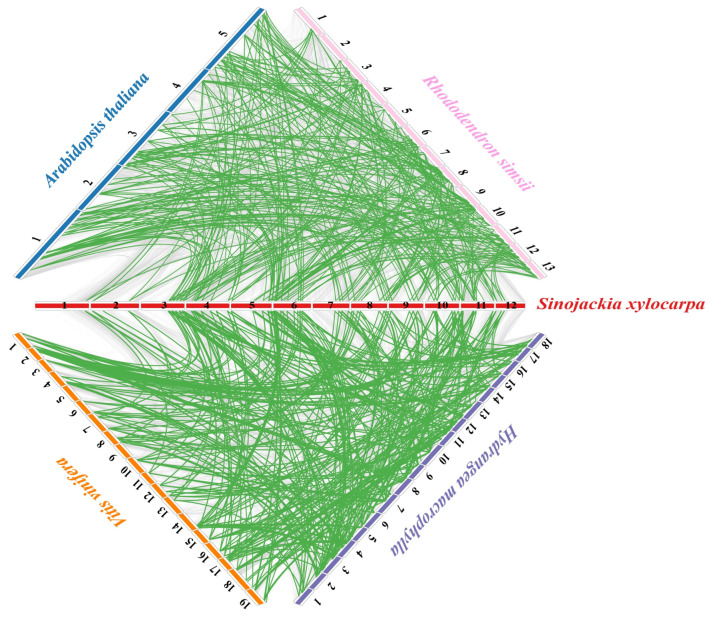
Comparative collinearity of *NAC* genes in *S. xylocarpa* and four representative dicot species. The genomes of *A. thaliana*, *R. simsii*, *H. macrophylla*, and *V. vinifera* are shown at the four corners, with individual chromosomes indicated by colored bars and numbers. The 12 chromosomes of *S. xylocarpa* are arranged along the central diagonal. Green curves connect collinear *NAC* gene pairs among the five species, while grey curves represent background whole-genome collinear gene pairs.

To further elucidate the evolutionary relationships of the *NAC* gene family, we constructed interspecific syntenic maps between *S. xylocarpa* and four other plant species—*Hydrangea macrophylla*, *Rhododendron simsii*, *V. vinifera* and *A. thaliana* (Figure 2). In total, 75, 71, 70 and 65 *SxyNAC* genes, respectively, exhibited syntenic relationships with *NAC* genes from *H. macrophylla*, *V. vinifera*, *R. simsii* and *A. thaliana*. We detected 159, 104, 120, and 115 homologous *NAC* gene pairs when *S. xylocarpa* was compared with the four species (in that order), indicating that a substantial proportion of collinear homologs have been retained across different dicot lineages and that the *SxyNAC* family is overall highly conserved. Detailed lists of these syntenic *NAC* gene pairs for each species are provided in Tables S8–S11. Notably, *S. xylocarpa* and *H. macrophylla* share the largest number of syntenic genes and homologous gene pairs, suggesting that these two species have preserved more ancestral NAC-containing chromosomal segments, have undergone comparatively fewer gene loss events and chromosomal rearrangements, and therefore display a closer evolutionary relationship at the *NAC* loci. Taken together, the extensive *NAC* synteny observed between *S. xylocarpa* and the four representative dicot species implies that most *SxyNAC* genes likely originated prior to the divergence of these lineages and were subsequently co-retained. Meanwhile, the variation in synteny levels and in the numbers of homologous gene pairs among species reflects lineage-specific histories of gene duplication, loss and genomic rearrangement, ultimately shaping the present, partially species-specific composition of *NAC* gene families.

### 2.3. Phylogenetic Classification and Subfamily Distribution of NAC Genes in S. xylocarpa

**Figure 3:**
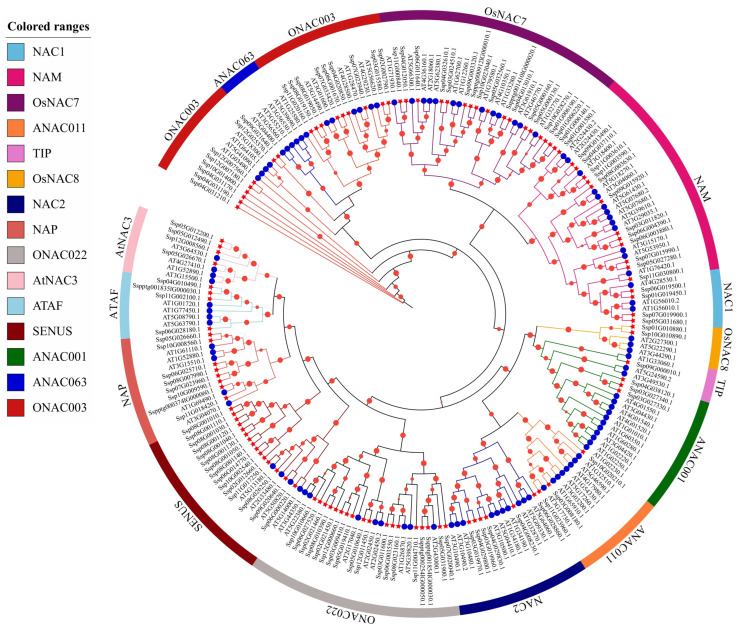
Neighbor-joining phylogenetic tree of NAC proteins from *S. xylocarpa* and *A. thaliana*. Red nodes represent *A. thaliana* NAC (AtNAC) proteins and blue nodes represent *S. xylocarpa* NAC (SxyNAC) proteins. The colored outer sectors indicate the 15 NAC subfamilies (NAC1, NAM, OsNAC7, ANAC011, TIP, OsNAC8, NAC2, NAP, ONAC022, AtNAC3, ATAF, SENUS, ANAC001, ANAC063 and ONAC003) defined according to Ooka et al., as shown in the legend on the left. Gene labels in the figure correspond to the original genome annotation IDs; the mapping to the custom IDs is provided in Table S1.

Based on multiple amino acid sequence alignments of NAC proteins from *A. thaliana* and *S. xylocarpa*, an ML tree with 1000 bootstrap replications was constructed to investigate the phylogenetic relationships of *SxyNAC* genes in *S. xylocarpa* (Figure 3). According to the classification system of the NAC family proposed by Hisako Ooka and colleagues [26], all NAC members were grouped into 15 subfamilies, namely NAC1, NAM, OsNAC7, ANAC011, TIP, OsNAC8, NAC2, NAP, ONAC022, AtNAC3, ATAF, SENUS, ANAC001, ANAC063 and ONAC003. Overall, most major clades showed moderate to high bootstrap support; however, the boundaries among some subfamilies were not sharply resolved, with a certain degree of intermingling and nesting observed between lineages, indicating that NAC subfamily delineation across species exhibits a continuum and some degree of ambiguity.

In the phylogenetic tree generated in this study, *SxyNAC* members are widely distributed across these subfamilies and cluster interspersedly with homologous NAC proteins from *A. thaliana*, reflecting a high level of sequence conservation and a shared evolutionary origin. Nevertheless, both the number and composition of members differ markedly among subfamilies. For example, *SxyNAC* members are enriched in the ONAC022, OsNAC7, ONAC003, and SENUS groups, implying that these branches likely experienced substantial duplication-driven expansion in *S. xylocarpa*, potentially associated with the specific requirements of woody plants in growth and development, stress responses or secondary growth. By contrast, some subfamilies contain only a few *SxyNAC* members. Notably, the ANAC063 subfamily, which was originally defined as a separate group in *A. thaliana*, does not form a fully independent monophyletic clade in our tree; instead, its members are interwoven with those of the ONAC003 branch. This pattern implies that ANAC063-type genes in *S. xylocarpa* may have experienced gene loss or have not undergone obvious expansion, thereby exhibiting lineage-specific characteristics. Several branches dominated by *SxyNAC* members can also be observed in the tree, suggesting that these gene groups have undergone species-specific duplication and expansion in *S. xylocarpa* and formed distinct evolutionary lineages, which may be closely associated with functional diversification related to its woody growth habit and adaptation to specific ecological environments.

### 2.4. Exon–Intron Organization and Conserved Motif Distribution of NAC Genes in S. xylocarpa

**Figure 4:**
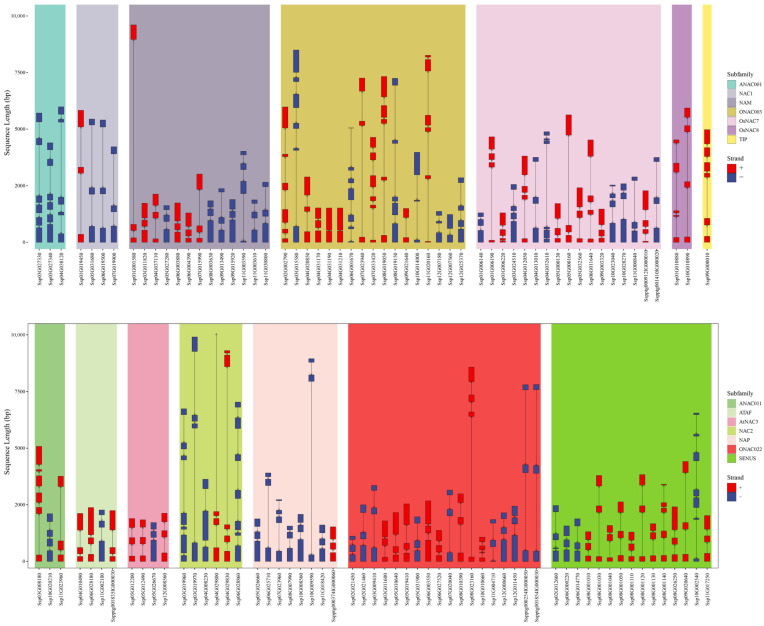
Gene structure organization of *NAC* family members grouped by subfamilies. The x-axis shows *NAC* gene IDs and the *y*-axis indicates sequence length (bp). Exons are shown as boxes and introns as connecting lines. Genes on the positive (+) and negative (−) strands are highlighted in red and blue, respectively. Shaded background blocks denote NAC subfamilies (e.g., ANAC001, NAC1, NAM, ONAC003, OsNAC7, OsNAC8, TIP, as well as ANAC011, ATAF, AtNAC3, NAC2, NAP, ONAC022, and SENUS). Gene labels in the figure correspond to the original genome annotation IDs; the mapping to the custom IDs is provided in Table S1.

To further characterise the *SxyNAC* genes, we examined their exon–intron structures (Figure 4 and Figure S2). The average exon number of each NAC subfamily is shown in Figure S3. Among all identified *SxyNAC* genes, 59 (51.3%) are transcribed from the sense strand (+), whereas 56 (48.7%) are transcribed from the antisense strand (−). The number of exons in *NAC* genes ranges from 2 to 7, corresponding to 1–6 introns, with an average of approximately 3.56 exons per gene. Some genes exhibit a compact structure with only a single intron, whereas others contain a larger number of introns and show markedly extended gene lengths. Both exon length and intron length, as well as intron spacing, vary substantially among genes, indicating that the *SxyNAC* family displays pronounced structural diversity at the gene level. *NAC* genes exhibited pronounced subfamily-specific structural divergence. Long genes with extended introns were relatively enriched in the NAC2 and ONAC003 subfamilies (e.g., *SxyNAC002*, *SxyNAC012*, *SxyNAC062* and *SxyNAC065*), whereas the ATAF and AtNAC3 subfamilies were characterized predominantly by compact gene architectures. Notably, exceptionally long members were observed in the NAM and NAP subfamilies (e.g., *SxyNAC035* and *SxyNAC105*, respectively), and ONAC022 also contained a markedly elongated gene (SxyNAC079) together with long-structure ptg genes (*SxyNAC083* and *SxyNAC084*). These patterns suggest that substantial structural expansion may have occurred in these loci and could be associated with subfamily-level diversification.

**Figure 5:**
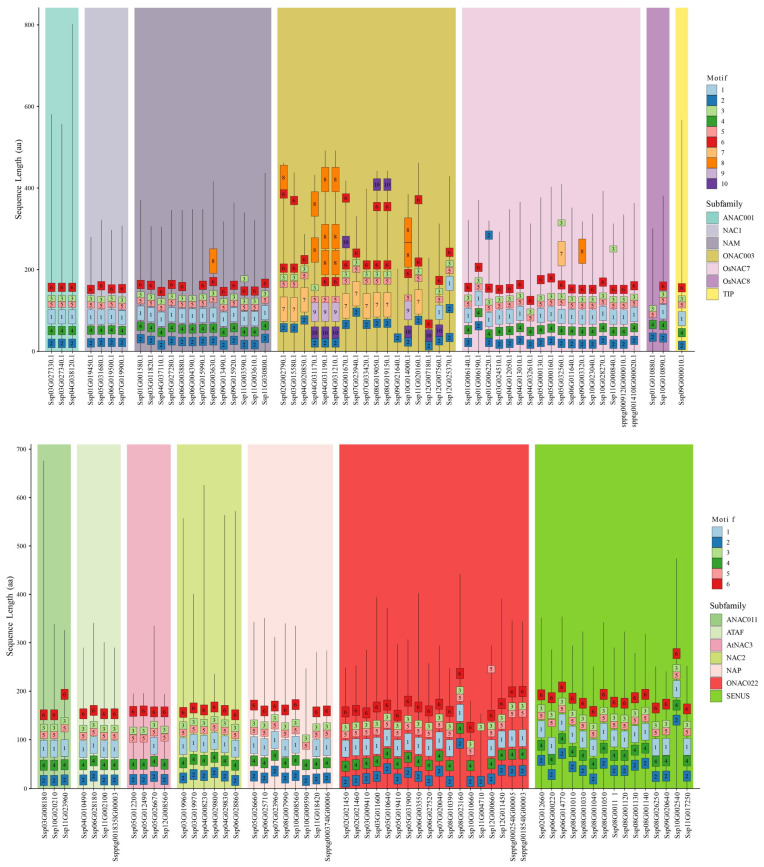
Conserved motif composition and protein length variation of SxyNAC members across subfamilies. The x-axis lists individual SxyNAC proteins (gene IDs) and the y-axis indicates protein sequence length (aa). Colored blocks represent conserved motifs, with colors and embedded numbers denoting motif types (see legend). Vertical stacking reflects the motif composition and arrangement within each protein. Shaded background panels delineate NAC subfamilies (see legend); the upper and lower panels display two sets of subfamilies. Gene labels in the figure correspond to the original genome annotation IDs; the mapping to the custom IDs is provided in Table S1.

To obtain a clearer view of structural diversity among *SxyNAC* genes, conserved motifs were identified across all NAC protein sequences using MEME, yielding a total of 10 Motifs (Figure 5 and Figure S4). The consensus sequences (sequence logos) of these motifs are shown in Figure S5. Notably, Motifs 1–6 were repeatedly detected at the N-terminal region in most NAC proteins, and their ordering pattern remained highly conserved.

According to Figure 5, the conserved motif distribution of SxyNACs shows a typical pattern of “N-terminal enrichment and C-terminal variability”. Motifs are largely concentrated toward the N-terminus; in contrast, the C-terminal portion tends to be extended to varying extents and contains comparatively few motifs, indicating that while the N-terminus maintains a relatively stable conserved framework, the C-terminus displays higher structural plasticity. Taking the ONAC003 subfamily as an example, its N-terminal motif arrangement exhibits clear subfamily-specific features: most members show a continuous pattern of Motif2–Motif7–Motif5–Motif3–Motif6, suggesting that ONAC003 proteins are largely consistent in their core motif composition and order. Meanwhile, a few members display evident motif rearrangement/substitution, characterized by the pattern Motif2–Motif10–Motif9–Motif5 (e.g., SxyNAC004, SxyNAC005, SxyNAC006, and SxyNAC013), implying that, besides the predominant architecture, another relatively stable motif configuration exists within ONAC003, reflecting structural diversity within this subfamily. Notably, several ONAC003 members exhibit more unusual motif combinations: for instance, SxyNAC012 contains only Motif 2; SxyNAC015 shows a simplified arrangement of Motif2–Motif10–Motif6; the N-terminus of SxyNAC016 is organized as Motif2–Motif10–Motif1–Motif5–Motif3–Motif6; and Sxy017 displays Motif2–Motif2–Motif1–Motif5–Motif3–Motif6, indicating varying degrees of motif loss, insertion, or duplication among ONAC003 members. In addition, Motif6, Motif8, and Motif10 are still detectable in the C-terminal regions of most ONAC003 proteins, further suggesting considerable variation within the non-conserved regions.

In contrast, other NAC subfamilies (e.g., OsNAC7, OsNAC8, NAM, NAC1, ANAC001, and TIP) tend to share a more uniform N-terminal core framework, most commonly arranged as Motif2–Motif4–Motif1–Motif5–Motif3–Motif6. Although a few members contain additional motifs beyond this core architecture (for example, some OsNAC7 proteins harbor Motif7 or Motif8, and certain NAM members also contain Motif8), the overall motif organization remains highly conserved. Of note, within the AtNAC3 subfamily, only SxyNAC114 exhibits the Motif2–Motif10–Motif9–Motif5 pattern, whereas the remaining members generally lack Motif 1 at the N-terminus, indicating subfamily-specific differences in N-terminal motif composition. As a supplementary analysis, conserved motifs were also analysed at the CDS level, and the corresponding motif distribution patterns are provided in Figure S6. Collectively, these results demonstrate that NAC proteins in this species possess relatively conserved motif composition and order at the N-terminus, with distinct motif architectures across subfamilies, while pronounced differences in C-terminal length and motif occurrence patterns reflect substantial structural divergence within the non-conserved regions.

### 2.5. Cis-Acting Element Composition in Promoter Regions of S. xylocarpa NAC Genes

**Figure 6:**
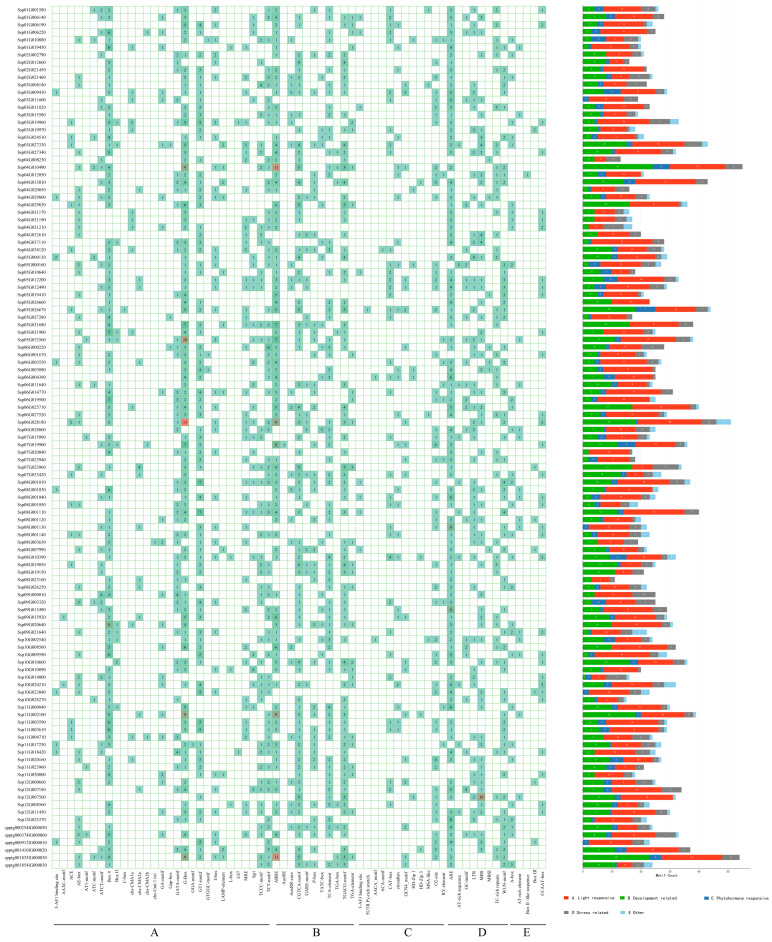
Promoter *cis*-element distribution and functional grouping for *SxyNAC* genes in *S. xylocarpa*. *Cis*-elements predicted in the upstream promoter segments (2000 bp relative to the transcription start site) of 115 *SxyNAC* genes were cataloged and assigned to five functional classes: (A) Light responsive, (B) Development related, (C) Phytohormone responsive, (D) Stress related, and (E) Other. The left heatmap shows the presence/absence and copy number of individual *cis*-elements in each *SxyNAC* promoter, and the numbers in the colored boxes indicate the counts of the corresponding *cis*-elements. The stacked bar charts on the right summarize, for each gene, the total number of *cis*-elements belonging to the five functional categories. Gene labels in the figure correspond to the original genome annotation IDs; the mapping to the custom IDs is provided in Table S1.

To investigate transcriptional control features of *SxyNAC* genes and explore their putative links to secondary-metabolite pathways, we surveyed *cis*-elements within promoter regions from 115 *NAC* genes (covering a 2 kb segment upstream of the transcription start point) and subsequently assigned the identified elements to functional categories (Figure 6, Table S12). Based on their putative functions, the detected *cis*-elements were classified into five major categories: light-responsive elements, development-related elements, phytohormone-responsive elements, stress-related elements and elements with other functions. Our analysis indicated that the promoter regions of nearly all *SxyNAC* genes contain diverse *cis*-element types, whereas the abundance and combination of these elements differ substantially across individual genes. The overall numbers of the *cis*-acting elements across *SxyNAC* promoters are summarised in Figure S7. As an illustration, the SxyNAC100 promoter harbors exclusively light- and development-associated elements, while hormone-responsive motifs are absent from SxyNAC078, pointing to highly specific cis-element combinations in some family members [27].

Light-responsive elements represented the most diverse category and also exhibited the highest total copy number across all genes. Among them, G-box, Box 4, and GT1-motif occurred with the highest frequency, suggesting that a large proportion of *SxyNAC* genes may be regulated by light signals and are potentially involved in photosynthetic metabolism and flavonoid biosynthesis. Light signaling has been demonstrated to promote anthocyanin and flavonoid biosynthesis by modulating transcriptional networks comprising NAC, MYB, and bHLH transcription factors, suggesting that certain *SxyNAC* genes could be involved in modulating fruit development and pigment deposition in *S. xylocarpa* [3,28].

Development-related elements were detected in a considerable proportion of *SxyNAC* promoters, although their overall abundance was lower than that of light-responsive elements. The relatively frequent development-related motifs included CAT-box, which is associated with tissue and organ differentiation, and O2-site, which is involved in the regulation of seed or storage protein expression. These elements displayed a markedly uneven distribution among genes, and are typically associated with tissue-specific expression as well as fruit or seed development. Notably, a subset of *SxyNAC* genes simultaneously carried multiple development-related elements together with hormone-responsive elements, suggesting that these members may contribute to the coordination between hormone signaling and the accumulation of metabolic products during the late stages of fruit development.

Within the phytohormone-related category, ABRE motifs linked to abscisic acid (ABA) signaling showed the highest occurrence, followed in frequency by the MeJA-associated CGTCA and TGACG motifs, and then by the salicylic acid (SA)–responsive TCA-element. The enrichment of ABRE elements implies that *SxyNAC* genes may be transcriptionally regulated by ABA signaling and thereby participate in fruit ripening and stress responses [6,29,30,31,32,33,34,35]. In many plant species, ABA has been shown to induce NAC and MYB transcription factors to cooperatively activate genes involved in anthocyanin, lignin, and defense-related secondary metabolic pathways [3,6,36,37,38,39,40,41]. The widespread presence of MeJA-responsive elements further suggests that *SxyNAC* genes may be involved in jasmonate-mediated defense responses and reprogramming of secondary metabolism, particularly in flavonoid and alkaloid biosynthesis and other antioxidant pathways [42,43,44,45]. The coexistence of multiple hormone-responsive elements within the same promoters indicates that the *SxyNAC* family may act as integrative nodes in the crosstalk among ABA–JA–SA signaling pathways that coordinate metabolic reconfiguration.

In addition, although the diversity of stress-related elements was relatively limited, their copy numbers in most *SxyNAC* promoters exceeded those of hormone-responsive elements. Typical stress-related motifs included ARE, the drought-responsive MBS (a MYB-binding site), the cold-responsive LTR element, and TC-rich repeats implicated in defense and stress responses. The prevalence of these elements in *SxyNAC* promoter regions points to a capacity for *SxyNAC* genes to perceive diverse environmental stresses (e.g., drought, cold, and pathogen challenge) and to contribute to *S. xylocarpa* adaptation through regulation of defense-associated secondary-metabolite pathways, including phenylpropanoid metabolism and lignin biosynthesis [46,47,48]. As a woody species, *S. xylocarpa* accumulates abundant secondary metabolites (e.g., phenolic acids, flavonoids, and lignin-related compounds) that play crucial roles in stress tolerance and antioxidation; therefore, NAC-mediated stress responses and regulation of metabolic pathways are likely to be of particular physiological significance in this species.

### 2.6. Expression Analysis of NAC Genes During Six Fruit Developmental Stages in S. xylocarpa

Field survey results showed that a total of 138 *S. xylocarpa* individuals were recorded, with tree height ranging from 1.1 to 8.3 m and DBH ranging from 1.3 to 180 cm. Based on this survey, fruit phenotypic changes during development were further analyzed. As fruit development progressed from April to September, the phenotypic traits of *S. xylocarpa* fruits also changed markedly. Fruit weight, length, and width all exhibited an overall increasing trend during development, although their dynamic patterns were not entirely consistent (Figure S8). Among the three traits, fruit weight displayed the most pronounced dynamic change, showing a continuous increase with stage-dependent acceleration. Statistical comparisons revealed a significant difference between April and May, indicating that fruit weight accumulation had already initiated rapidly at the early developmental stage. The difference between April and June became more pronounced, and a significant difference was also observed between June and July, suggesting that June to July represents a critical period of rapid fruit weight gain. In addition, comparisons between May and July, April and July, May and August, June and August, and April and August/September all reached extremely significant levels, further demonstrating that the fruits underwent marked weight gain during the early-to-middle developmental stages and maintained a relatively high level thereafter.

Compared with fruit weight, fruit length changed more gradually during development, with a relatively limited amplitude of increase. Significant differences were observed between April and May and became more pronounced between April and June, indicating that longitudinal elongation had already occurred at the early stage. The differences between April and July and between April and August reached extremely significant levels, suggesting that the increase in fruit length mainly occurred during the early-to-middle stages of development. A significant difference was still detected between April and September, indicating that although fruit length changed relatively little at the late stage, it remained overall higher than at the initial stage. Significant differences between May and several later developmental stages also suggest that fruit length continued to increase after May, although the magnitude of change was less pronounced than that of fruit weight and width.

The trend in fruit width was intermediate between those of fruit weight and fruit length, but its growth pattern was more directly associated with fruit enlargement. Significant differences were observed between April and June, indicating that fruit width had already increased markedly during the early stage. A significant difference was also detected between May and July, suggesting that May to July was an important period of rapid fruit width expansion. Moreover, the differences between April and July, August, and September all reached extremely significant levels, indicating that fruit width had increased substantially by the middle and late stages compared with the initial stage. Significant differences between May and August and between May and September further support the conclusion that fruit width expansion was mainly concentrated in the early-to-middle stages, whereas changes became relatively minor at the later stage.

**Figure 7:**
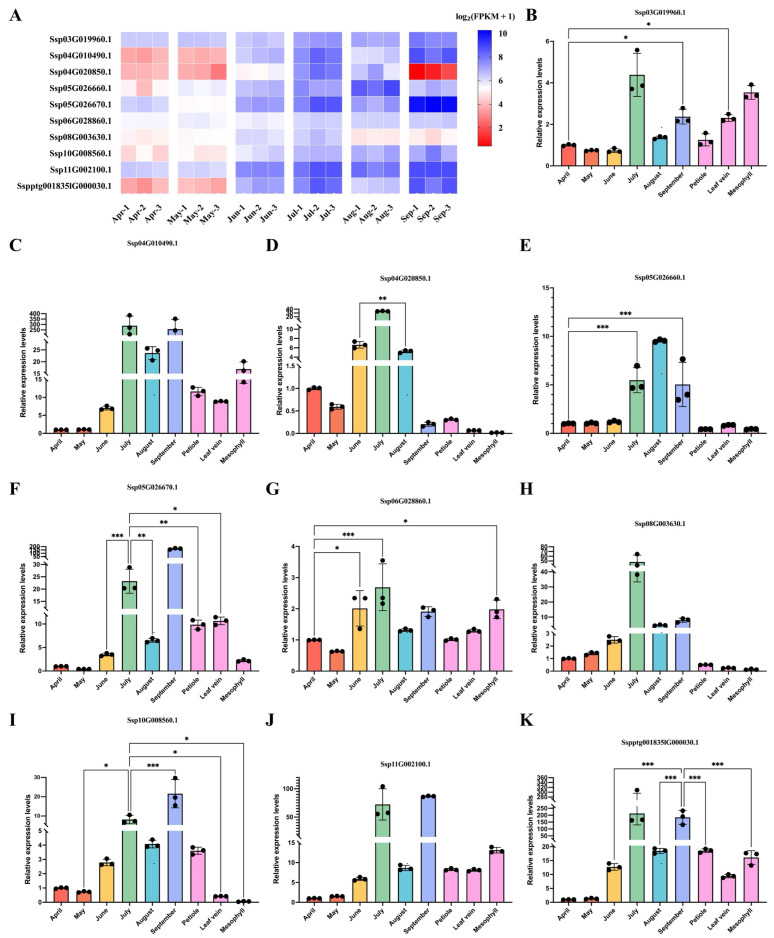
Expression dynamics of ten candidate genes during fruit development (April–September) and in leaf tissues. (**A**) RNA-seq-based heatmap depicting transcript abundance patterns of the ten candidates in fruit samples harvested between April and September; colors indicate expression levels as log_2_(FPKM + 1). Three biological replicates were included for each month (Apr-1–3, May-1–3, …, Sep-1–3). (**B**–**K**) qRT–PCR analysis of the relative expression levels of the indicated genes in fruits collected at different months (April–September) and in leaf tissues (petiole, leaf vein, and mesophyll): *SxyNAC061* (**B**), *SxyNAC108* (**C**), *SxyNAC003* (**D**), *SxyNAC100* (**E**), *SxyNAC114* (**F**), *SxyNAC066* (**G**), *SxyNAC042* (**H**), *SxyNAC104* (**I**), *SxyNAC110* (**J**), *SxyNAC111* (**K**). Bars represent mean ± SD (*n* = 3), with black dots indicating biological replicates. Broken axes were used in selected panels to display measurements spanning multiple orders of magnitude. Brackets and asterisks indicate statistically significant differences (one-way ANOVA with Tukey’s multiple-comparisons test): * *p* < 0.05, ** *p* < 0.01, *** *p* < 0.001. qRT–PCR measurements were normalized against a housekeeping gene, and relative transcript levels were derived via the 2^−ΔΔCt^ approach. Gene labels in the figure correspond to the original genome annotation IDs; the mapping to the custom IDs is provided in Table S1.

Based on transcriptome data from six fruit developmental stages (F1–F6, April to September), we systematically characterized the expression profiles of the *NAC* gene family in *S. xylocarpa* during fruit development (Figure 7A). RNA sequencing of *S. xylocarpa* fruits across six developmental stages was performed using 18 samples. In total, 457.12 million raw reads and 114.75 Gb clean data were obtained. Among the 115 identified *SxyNAC* genes, 88 were included in the transcriptomic expression analysis. The RNA-seq expression matrix [log_2_(FPKM + 1)] for all stages with three biological replicates is provided in Table S13. Overall, the majority of *NAC* genes showed detectable expression across multiple developmental stages, indicating that members of this family may participate in fruit growth and development at different phases.

To assess the robustness of the RNA-seq dataset and identify key candidates, ten representative *NAC* genes were selected based on transcript abundance and fold change. Their expression levels were further quantified by qRT-PCR in fruits across the six developmental stages and in September leaf tissues (petiole, vein, and mesophyll) (Figure 7B–K). The qRT-PCR results revealed pronounced stage-dependent expression differences among these ten *NAC* genes during fruit development, as well as distinct tissue preferences among leaf tissues in September. In general, most genes were upregulated at the middle-to-late stages of fruit development, particularly in July or September, whereas several genes exhibited extremely low expression in leaf tissues, suggesting functional differentiation among *NAC* family members. In addition, the expression trajectories of most genes across the six fruit stages were largely consistent with the RNA-seq patterns, thereby supporting the reliability of the transcriptome-derived expression profiles.

Further integration of the qRT-PCR data with fruit phenotypic changes revealed clear temporal associations between candidate *NAC* gene expression and fruit development. *SxyNAC061*, *SxyNAC003*, *SxyNAC042*, and *SxyNAC066* reached high expression levels or showed relative peaks in July. These expression peaks closely coincided with the period when fruit weight increased most rapidly and transverse expansion was most pronounced, suggesting that these genes may be associated with rapid fruit enlargement or related developmental processes during the middle stage. *SxyNAC108* began to be upregulated in June and maintained relatively high expression levels in both July and September, suggesting that it may participate in both middle-stage enlargement and late-stage developmental regulation. In contrast, *SxyNAC100* and *SxyNAC104* reached relatively high expression levels in August or September, when the external fruit morphology had largely stabilized while fruit weight continued to accumulate, indicating that these genes may be more closely associated with late-stage fruit filling, maturation, or metabolite accumulation. In addition, *SxyNAC114*, *SxyNAC110*, and *SxyNAC111* maintained relatively high expression levels from July to September, spanning both the rapid fruit enlargement stage and the subsequent filling stage, suggesting that they may play sustained regulatory roles during the middle and late stages of fruit development. Overall, the expression dynamics of these candidate *NAC* genes showed good temporal consistency with the phenotypic changes in fruit development, indicating that they may play functionally differentiated regulatory roles during fruit enlargement, filling, and maturation.

To further investigate tissue-specific expression patterns, we compared the transcript abundance of the selected *NAC* genes among different leaf tissues. In September leaf samples, *SxyNAC061* peaked in the mesophyll, showed intermediate expression in the veins, and was lowest in the petiole, indicating a pronounced tissue-dependent gradient. In contrast, *SxyNAC003*, *SxyNAC100*, and *SxyNAC042* exhibited extremely low transcript abundance in all three leaf tissues, representing a leaf low-expression pattern. *SxyNAC114* showed significantly higher expression in the petiole and leaf vein than in the mesophyll, indicating a preference for vascular or connecting tissues. Similarly, *SxyNAC104* was relatively more abundant in the petiole, whereas its expression in the leaf vein and mesophyll remained low, consistent with a petiole-preferential distribution.

A further comparison between September fruits and leaf tissues collected at the same time point showed that *SxyNAC042*, *SxyNAC100*, *SxyNAC104*, *SxyNAC108*, *SxyNAC110*, *SxyNAC111*, and *SxyNAC114* exhibited higher expression levels or apparent enrichment in fruits relative to leaf tissues. By contrast, *SxyNAC061* and *SxyNAC066* did not show a clear fruit-enriched pattern in September: the former displayed relatively higher expression in leaf tissues, particularly in the mesophyll, whereas the latter showed comparable transcript abundance in the mesophyll and fruits. Notably, *SxyNAC003* remained at extremely low expression levels in both September fruits and all three leaf tissues, with no evident organ- or tissue-specific high expression at this stage.

## 3. Discussion

We identified 115 *NAC* genes from the *S. xylocarpa* genome in this work. This family size is comparable to that reported for most woody species. It is slightly higher than that of the herbaceous species *O sativa* (75) and the woody perennial species *V. vinifera* (74) [26,49], but lower than that of *Populus trichocarpa* (170) and *Pyrus pyrifolia* (185) [50,51], indicating that the *SxyNAC* family has undergone a moderate degree of expansion in *S. xylocarpa*. Collinearity analyses revealed that this expansion was predominantly driven by whole-genome and segmental duplications, whereas tandem duplication contributed relatively little. This pattern is consistent with the widespread occurrence of genome duplication events in woody plants and points to the possibility that, in *S. xylocarpa*, the NAC family has evolved greater complexity in metabolic regulation via duplication events coupled with subsequent functional diversification. WGD-driven expansion of *NAC* gene families has likewise been documented in several other woody and herbaceous plant species, including *P. trichocarpa*, *Zea mays* and *Juglans regia* [50,52,53].

Based on *Ks* estimates and inferred divergence times, duplicated *SxyNAC* gene pairs span a broad temporal range from approximately 1.221 to 252.546 Mya and exhibit a clear multi-peak, multi-phase distribution. A subset of pairs with very small *Ks* values corresponds to divergence times of only a few million years, indicating that *S. xylocarpa* has continued to experience recent tandem or segmental duplication events after the establishment of its lineage. The majority of duplicated pairs (around 60%) fall within the 30–100 Mya window, which likely coincides with periods of rapid diversification at the order (*Ericales*) or family/genus level, representing a major expansion phase of the NAC family. In addition, roughly one-third of the duplicated pairs show divergence times exceeding 100 Mya, with some approaching or surpassing 200 Mya, implying that a set of ancient duplicate copies originating from early duplication events have been retained over long evolutionary timescales rather than being completely purged [19]. This “ancient duplication + intermediate expansion + recent duplication” temporal pattern is congruent with the evolutionary trajectories reported for NAC and other large gene families in many woody plants, in which early polyploidy events establish the core repertoire of family members, and subsequent segmental/tandem duplications further superimpose lineage-specific copies and functions. Taken together, the expansion of the *SxyNAC* family clearly spans multiple evolutionary stages before and after the divergence of the *S. xylocarpa* lineage, suggesting that this family not only participated in the adaptive diversification associated with early angiosperms or *Ericales*, but has also continued to experience novel selective pressures during later speciation and ecological radiation, ultimately shaping its present-day size and composition [54].

Phylogenetic analysis indicated that *SxyNAC* members could be classified into the 15 classical subfamilies proposed by Ooka et al. [26] and interspersed with *A. thaliana* NAC proteins. Overall, this reflects the high conservation and common origin of the NAC family in dicots. This recurring “subgroup framework conservation” in angiosperms is generally interpreted as evidence that major NAC lineages were established early, followed by continuous evolution through duplication–retention–divergence events across different lineages [1]. However, the distribution of *SxyNAC* members among these subfamilies was notably uneven: lineages such as ONAC022, OsNAC7, ONAC003, and SENUS contained a larger number of *S. xylocarpa* members, suggesting that these branches underwent significant expansion associated with the woody growth habit. Notably, the OsNAC7 subgroup contains the core transcriptional switches *SND1* (AT1G32770) and *NST1* (AT2G46770), which regulate secondary wall synthesis in *A. thaliana*. The specific expansion of *SxyNAC*s within this clade exemplifies the evolutionary strategy of woody plants in maintaining robust xylem development [55,56,57]. Conversely, some subfamilies (e.g., ANAC063) contained fewer members or were interwoven with other lineages, which may be attributable to gene loss or restricted expansion within the *S. xylocarpa* lineage.

Based on RNA-seq data from six fruit development stages (April to September), we screened 10 representative *SxyNAC* genes from *S. xylocarpa*. We validated their relative expression trends across the six fruit developmental stages using qRT-PCR and preliminarily analyzed their expression patterns in leaf vein, petiole, and mesophyll tissues in September. An enrichment of light-responsive and development-related *cis*-elements was evident across the promoters of *SxyNAC* genes; however, distinct biases in element composition were observed among different genes. *SxyNAC003*, *SxyNAC042*, *SxyNAC061*, and *SxyNAC066* exhibited the highest proportion of light-responsive elements, whereas *SxyNAC108* was most abundant in development-related elements. Additionally, *SxyNAC104*, *SxyNAC110*, *SxyNAC111*, and *SxyNAC114* showed simultaneous enrichment of both light-responsive and development-related elements, suggesting a potential regulatory mechanism that integrates environmental light signals with organ development programs.

Regarding expression patterns, among the genes dominated by light-responsive elements, *SxyNAC061* displayed a “mesophyll > vein > petiole” gradient in September leaves and significant upregulation during the mid-fruit development stage (July), reflecting a tissue preference associated with photosynthetic functional zones (mesophyll). Similarly, *SxyNAC066* maintained relatively high expression levels in the mesophyll and showed an upward trend during mid-fruit development. Together, these findings suggest that the expression of these genes is likely related to leaf photosynthetic/metabolic status and changes synchronously with the fruit development process. It is worth noting that *SxyNAC003* and *SxyNAC042*, which are also dominated by light-responsive elements, exhibited significant induction during mid-fruit development (especially in July) but maintained extremely low expression levels across all three leaf tissues in September. This pattern is characterized by “high expression in fruit stages versus low expression in leaves”, implying that the presence of light-responsive cis-elements does not strictly correlate with strong expression in leaf tissues.

By contrast, *SxyNAC108*, which possessed the most abundant development-related elements, exhibited a drastic upregulation during the mid-to-late fruit development stages (maintaining high levels in both July and September), while maintaining moderate expression in leaf tissues. This demonstrates a general trend of enrichment in mature fruit, which is consistent with the higher frequency of development-related *cis*-elements in its promoter, suggesting a closer association with transcriptional regulation of fruit development and ripening. According to previous studies, NAC family members in various fruit crops often occupy key hierarchical positions in ripening regulatory networks, influencing traits such as softening, pigmentation, and flavor-related metabolism [58,59,60]. Therefore, SxyNAC108 can be considered a priority candidate gene for the regulation of fruit ripening in *S. xylocarpa*.

As strong sink organs, mature fruits exhibit significantly increased demands for the unloading and accumulation of assimilates such as sugars, while petioles and veins serve as critical channels for source output and long-distance transport. Existing reviews have systematically summarized the core role of source–sink interactions and assimilate partitioning in fruit growth and ripening; direct evidence also demonstrates that certain NACs can regulate sugar transport-related genes, thereby influencing sugar accumulation at the sink end. In agreement with these results, *SxyNAC114* showed markedly higher transcript levels in the petiole and vein than in the mesophyll, suggesting a clear preference for vascular-associated leaf tissues. This suggests that the gene is more likely related to transport tissue function or source–sink substance/signal transmission rather than primarily functioning in mesophyll photosynthesis. This inference aligns with the current understanding of NAC functions: extensive research indicates that NACs (particularly the VND/NST/SND modules) act as “upstream switches” in xylem/vascular differentiation, secondary wall formation, and cell wall-related gene networks, driving the transcriptional activation of cellulose and lignin pathways to influence vascular tissue formation and function [61,62]. Additionally, *SxyNAC104* showed relatively higher expression in petioles, while *SxyNAC110* and *SxyNAC111* reached high expression levels in September fruit while maintaining detectable expression in leaf tissues. These expression characteristics suggest that these genes may simultaneously respond to developmental programs and light-related signals during the maturation phase, establishing a certain expression coupling between leaf transport-related tissues (petiole/vein) and the fruit ripening process, thereby likely participating in the coordinated regulation between source and sink [63].

## 4. Materials and Methods

### 4.1. Genome Data Retrieval and Identification of NAC Genes in Sinojackia xylocarpa

The nuclear reference genome of *S. xylocarpa* (GWH/NGDC no. GWHCBFM00000000) used in this study was obtained from our previously reported chromosome-level, high-quality nuclear genome assembly of this species, which serves as the model species of the genus *Sinojackia* [18]. The reference genome sequence, CDS files and annotation files for *H. macrophylla* ‘Endless Summer’ were downloaded from Data.gov—the U.S. Government’s Open Data portal (https://data.gov, accessed on 10 July 2025). Reference genome sequences, protein sequences and GFF annotation files for *R. simsii* (accession no. GCA_047301955.1) and *V. vinifera* (accession no. GCF_030704535.1) were downloaded via NCBI (https://www.ncbi.nlm.nih.gov/, accessed on 2 April 2025) [64,65,66]. NAC protein sequences for *A. thaliana* were sourced from TAIR (https://www.arabidopsis.org/, accessed on 2 April 2025) [67]. Identification of NAC proteins in *S. xylocarpa* was initiated with a profile-based search: the complete predicted proteome was queried in HMMER (v3.4) using the NAM-domain model PF02365.15, retaining hits with E ≤ 1 × 10^−3^ [68]. The retained sequences were next evaluated by BLASTp (v2.16.0+) against the *A. thaliana* NAC (AtNAC) protein collection, applying E ≤ 1 × 10^−5^ [69]. Domain confirmation was performed through Pfam (http://pfam.xfam.org/) to ensure that each candidate contained the conserved NAC core region. Physicochemical indices for all SxyNAC proteins—instability index, aliphatic index, GRAVY, GC content, pI, amino-acid length, and molecular weight (MW)—were computed with Biopython (v1.85) [70].

### 4.2. Multiple Alignment of Sequences and Phylogenetic Tree Construction

ClustalW (v2.1) was applied to align the SxyNAC protein sequences under default settings [71]. Model selection was carried out in IQ-TREE2 (v2.3.6) with ModelFinder, which chose JTT + F + R7 as the top-ranked substitution model under the lowest Bayesian information criterion (BIC). Maximum-likelihood (ML) phylogenetic trees were then constructed in IQ-TREE2 (v2.3.6) under this model with 1,000 bootstrap replicates [72,73]. The resulting ML tree was displayed via the iTOL web server (https://itol.embl.de/) [74].

### 4.3. Gene Structure and Conserved Motif Analysis

The exon–intron architecture of *SxyNAC* genes was characterized using the *S. xylocarpa* genome assembly together with its GFF3 annotation files. Gene structure diagrams were generated in R (v4.5.2, https://www.r-project.org/, accessed on 12 December 2025) with the ggplot2 package (v3.5.2, https://github.com/tidyverse/ggplot2, accessed on 29 December 2025). Conserved motifs within SxyNAC proteins were identified using the MEME Suite (v5.5.8, https://meme-suite.org/, accessed on 29 December 2025) [75]. MEME was run with the motif width set to 6–50 amino acids and the maximum number of motifs fixed at 15.

### 4.4. Chromosomal Mapping and Duplication Pattern Analysis

Chromosomal coordinates of *NAC* genes were obtained from the *S. xylocarpa* GFF annotation and visualized in TBtools2 (v2.376) [76]. For comparative analyses, genome sequences and corresponding GFF annotations for four dicot species (*H. macrophylla*, *R. simsii*, *V. vinifera*, and *A. thaliana*) were pairwise compared with *S. xylocarpa* in TBtools2 to generate interspecific synteny files [76]. Collinearity between *S. xylocarpa* and the four comparison species was visualized with the R package RIdeogram (v0.2.2; https://cran.r-project.org/web/packages/RIdeogram/, accessed on 12 August 2025) [77]. To examine evolutionary patterns within the *SxyNAC* family, *Ka* and *Ks* values were calculated for duplicated *SxyNAC* gene pairs using the *KaKs*-Calculator module in TBtools2 (v2.376) [76].

### 4.5. Promoter cis-Element Analysis Using PlantCARE

For each *SxyNAC* gene, a 2 kb promoter segment upstream of the translation initiation codon (ATG) was retrieved from the *S. xylocarpa* genome using TBtools2 (v2.376) [76]. PlantCARE (http://bioinformatics.psb.ugent.be/webtools/plantcare/html/, accessed on 21 August 2025) was used to screen these promoter sequences for *cis*- elements [27]. Identified elements were then categorized by functional type and displayed using in-house Python3 scripts (v3.12.3).

### 4.6. Subcellular Localization Prediction

Subcellular localization of SxyNAC proteins was assessed by separately submitting SxyNAC amino-acid sequences to BUSCA (https://busca.biocomp.unibo.it/, accessed on 9 August 2025) and WoLFPSORT (https://wolfpsort.hgc.jp/, accessed on 9 August 2025). The predicted localization results generated by the two tools were cross-compared to enhance the robustness of the prediction. All prediction outputs were compiled and are presented in Table S14.

### 4.7. RNA-Seq Dataset Preparation and Differential Expression Analysis

RNA sequencing of *S. xylocarpa* fruits spanning six developmental stages was carried out by Biomarker Technologies (Beijing, China). RNA-seq libraries were PCR-enriched, size-selected (~450 bp), quantified, pooled in equimolar proportions, and diluted to 2 nM. Raw-read quality was inspected with FastQC (v0.12.1). Adapter sequences were removed and low-quality reads were filtered out, i.e., reads with >10% N bases or >50% bases with Phred quality ≤ 10. After trimming, clean reads were mapped to the *S. xylocarpa* reference genome using HISAT2 (v2.2.1) [78]. Differential expression was evaluated with DESeq2 (v1.46.0) [79], applying *p* < 0.01 as the significance criterion. Heatmaps were produced with in-house Python scripts (v3.12.3). The RNA-seq dataset is available in the Genome Sequence Archive (GSA) at the National Genomics Data Center (NGDC), China National Center for Bioinformation (CNCB), under accession CRA016108 (https://ngdc.cncb.ac.cn/gsa/browse/CRA016108, accessed on 5 June 2025).

### 4.8. Plant Survey, Fruit Sampling, and Phenotypic Measurements

A total of 138 *S. xylocarpa* individuals were surveyed in the present study. Tree height and diameter at breast height (DBH) were measured for each individual, with DBH consistently assessed at 1.3 m above ground level. For multi-stemmed individuals, the diameter of each stem was measured separately at breast height and summed to obtain the total DBH for each individual. Based on the survey, five trees were selected for monthly fruit sampling from April to September. One fruit per tree was collected each month for measurements of fruit height, width, and weight. For qRT-PCR analysis, three samples from each monthly collection were used as biological replicates.

### 4.9. RNA Extraction and qRT-PCR Analysis

RNA samples were prepared from three leaf tissues (mesophyll, petiole, and vein) as well as fruits of *S. xylocarpa* using the SteadyPure Plant RNA Extraction Kit (Accurate Biology, Changsha, China). RNA quantity and integrity were checked by NanoDrop-based UV–visible spectrophotometry (Allsheng, Hangzhou, China) together with 1% agarose gel electrophoresis. First-strand cDNA was generated from purified RNA using the HiScript^®^ III 1st Strand cDNA Synthesis Kit (+gDNA wiper) (Vazyme, Nanjing, China; Cat. No. R312), and the synthesized cDNA was subsequently used for PCR and qRT-PCR. Amplicon specificity for the selected *SxyNAC* targets was confirmed by gel electrophoresis, with representative bands shown in Figure S9.

To evaluate candidate reference genes, fruits representing six developmental stages and leaves sampled in September were analyzed by qRT-PCR with three biological replicates per sample. Ct datasets were subjected to stability assessment using the comparative ΔCt approach, geNorm, BestKeeper, and NormFinder [80,81,82,83]. Outputs from these methods were combined to determine the most stable reference gene, and the EF1α homolog was chosen for normalization in subsequent qRT-PCR experiments.

Primer pairs were designed from *S. xylocarpa* CDS sequences in SnapGene (v6.0.2) [84], and all primer information is provided in Tables S15 and S16. qRT-PCR reactions were run on an Applied Biosystems StepOnePlus™ Real-Time PCR System (Applied Biosystems, Thermo Fisher Scientific, Waltham, MA, USA) using SupRealQ Ultra Hunter SYBR qPCR Master Mix (U+) (Vazyme, Nanjing, China; Cat. No. Q713). The program comprised 95 °C for 30 s, followed by 40 cycles of 95 °C for 10 s and 60 °C for 30 s; melt-curve analysis was performed from 60 °C to 95 °C with a 0.3 °C s^−1^ ramp and continuous fluorescence recording. Relative transcript abundance was derived with the 2^−ΔΔCt^ method [85], and each sample included three biological replicates.

## 5. Conclusions

We conducted a genome-wide survey of NAC transcription factors in *S. xylocarpa*, identifying 115 *SxyNAC* genes that encode the conserved NAC domain. These genes were systematically analysed with respect to their physicochemical properties, chromosomal distribution, phylogenetic classification, gene duplication patterns, intra- and interspecific collinearity, and expression profiles during fruit development. Integrative analysis of promoter cis-acting elements together with fruit development- and metabolism-related expression patterns suggested that a subset of *SxyNAC* genes may participate in the regulation of fruit growth, development, and metabolic processes in *S. xylocarpa.* Based on RNA-seq data from fruits collected between April and September, we further selected 10 representative *SxyNAC* genes and validated their expression characteristics in different tissues, including veins, petioles and mesophyll, of leaves sampled in September. The results revealed pronounced tissue specificity and developmental stage–dependent expression patterns. Collectively, these findings provide important candidates and foundational information for the further functional characterization of *NAC* genes involved in fruit development and metabolic regulation in *S. xylocarpa*, and lay a basis for the molecular improvement of related horticultural and agronomic traits.

## Data Availability

Data are contained within the CNCB.

## Supplementary Materials

The following supporting information can be downloaded at: https://www.mdpi.com/article/10.3390/plants15081163/s1, Figure S1: Chromosome-level localization of *SxyNAC* genes across the *S. xylocarpa* genome; Figure S2: Phylogenetic clustering and gene-structure features of *SxyNAC* genes; Figure S3: Average exon numbers of *SxyNAC* genes among different NAC subfamilies; Figure S4: Phylogenetic tree and conserved motif distribution of SxyNAC proteins; Figure S5: Sequence logos of conserved motifs identified in SxyNAC proteins (Motifs 1–10); Figure S6: Conserved motif architectures of *SxyNAC* genes based on CDS sequences; Figure S7: Frequency distribution of *cis*-acting elements in the promoter regions of *SxyNAC* genes; Figure S8: Changes in fruit phenotypic traits during development from April to September in *S. xylocarpa*. (A) fruit length, (B) fruit width, and (C) fruit weight at six developmental stages. Data are presented as mean ± SD. Statistical significance among stages was determined by one-way ANOVA followed by multiple comparisons. Asterisks indicate significant differences (* *p* < 0.05, ** *p* < 0.01, *** *p* < 0.001, **** *p* < 0.0001); Figure S9: Agarose gel electrophoresis image of PCR amplicons for selected *SxyNAC* genes; Table S1: Mapping of original gene IDs to custom gene IDs used in this study; Table S2: Physicochemical properties of SxyNAC proteins, including amino acid length, molecular weight, isoelectric point (pI), GC content, GRAVY, aliphatic index, and instability index; Table S3: Chromosomal locations of *SxyNAC* genes in the *S. xylocarpa* genome; Table S4: Distribution of NAC-enriched clusters across chromosomes in *S. xylocarpa*; Table S5: Classification of duplication types of *SxyNAC* genes; Table S6: Collinear gene pairs identified among *SxyNAC* genes in the *S. xylocarpa* genome; Table S7: *Ka, Ks, Ka/Ks* ratio, and divergence time (T) of homologous gene pairs; Table S8: Syntenic pairs between *S. xylocarpa* and *H. macrophylla* identified by comparative synteny analysis; Table S9: Syntenic pairs between *S. xylocarpa* and *V. vinifera* identified by comparative synteny analysis; Table S10: Syntenic pairs between *S. xylocarpa* and *A. thaliana* identified by comparative synteny analysis; Table S11: Syntenic pairs between *S. xylocarpa* and *R. simsii* identified by comparative synteny analysis; Table S12: *Cis*-acting regulatory elements identified in the promoter regions of *SxyNAC* genes, including their putative functions, functional categories, and occurrence numbers; Table S13: Expression profiles of selected *SxyNAC* genes across different samples based on RNA-seq data. Values are presented as log_2_(FPKM + 1); Table S14: Summary of *SxyNAC* genes and predicted subcellular localization; Table S15: Primer sequences (5′–3′) used for qRT-PCR analysis; Table S16: Primer sequences (5′–3′) used for PCR amplification.

## Abbreviations

The following abbreviations are used in this manuscript:

NAC	NAM, ATAF1/2 and CUC2
NAM	No Apical Meristem
ATAF1/2	Arabidopsis Transcription Activation Factor 1/2
CUC2	CUP-SHAPED COTYLEDON 2
qRT-PCR	quantitative real-time polymerase chain reaction
TRD	transcriptional regulatory domain
DNA	deoxyribonucleic acid
DBD	DNA-binding domain
CHS	chalcone synthase
F3H	flavanone 3-hydroxylase
DFR	dihydroflavonol 4-reductase
JUB1	JUNGBRUNNEN1
SA	salicylic acid
ABA	abscisic acid
bZIP	basic leucine zipper
MdUFGT	Malus domestica UDP-glucose:flavonoid 3-O-glucosyltransferase
FaRIF	Fragaria × ananassa RIPENING-INDUCED FACTOR
NOR	NON-RIPENING
PAL2	phenylalanine ammonia-lyase 2
JA	jasmonic acid
TIAs	terpenoid indole alkaloids
GA	gibberellin
PFAM	Protein families database
ID	identifier
aa	amino acid
Da	dalton
pI	isoelectric point
GC	guanine–cytosine (content)
GRAVY	grand average of hydropathicity
Chr	Chromosome
WGD	whole-genome duplication
Ka	nonsynonymous substitution rate
Ks	synonymous substitution rate
Mya	million years ago
T	divergence time
λ	substitution rate (substitutions/site/year)
ML	maximum likelihood
MEME	Multiple EM for Motif Elicitation
bp	base pair(s)
kb	kilobase(s)
G-box	G-box cis-acting element
GT1-motif	GT1 motif / GT-1 transcription factor binding site
MYB	MYB transcription factor family
bHLH	basic helix–loop–helix
CAT-box	CAT-box cis-acting element
O2-site	Opaque2 binding site
ABRE	abscisic acid responsive element
MeJA	methyl jasmonate
CGTCA-motif	CGTCA motif (MeJA-responsive cis-element)
TGACG-motif	TGACG motif (MeJA-responsive cis-element)
TCA-element	TCA element (salicylic acid-responsive cis-element)
ARE	anaerobic induction responsive element
MBS	MYB binding site (drought-inducible)
LTR	low-temperature responsive element
TC-rich repeats	thymine–cytosine rich repeats
SND1	SECONDARY WALL-ASSOCIATED NAC DOMAIN PROTEIN 1
NST1	NAC SECONDARY WALL THICKENING PROMOTING FACTOR 1
VND	VASCULAR-RELATED NAC-DOMAIN
NST	NAC SECONDARY WALL THICKENING PROMOTING FACTOR
SND	SECONDARY WALL-ASSOCIATED NAC DOMAIN
GWH	Genome Warehouse
NGDC	National Genomics Data Center
CDS	coding sequence
GFF	General Feature Format
NCBI	National Center for Biotechnology Information
TAIR	The Arabidopsis Information Resource
HMM	hidden Markov model
BLASTp	Basic Local Alignment Search Tool (protein)
MW	molecular weight
JTT+F+R7	Jones–Taylor–Thornton model + empirical frequencies (+F) + FreeRate model with 7 categories (R7)
BIC	Bayesian information criterion
GFF3	General Feature Format version 3
PlantCARE	Plant Cis-Acting Regulatory Elements database
DEGs	differentially expressed genes
UV	ultraviolet
cDNA	complementary DNA
gDNA	genomic DNA
PCR	polymerase chain reaction
Ct	cycle threshold
EF1α	elongation factor 1 alpha
