# Supplementary figures and images for: Genome-Wide Identification and Characterization of the NAC Transcription Factor Family in Sinojackia xylocarpa Hu
Source: Plants (Basel). 2026 Apr 9;15(8):1163. doi: 10.3390/plants15081163 (PMC13119869; doi:10.3390/plants15081163)

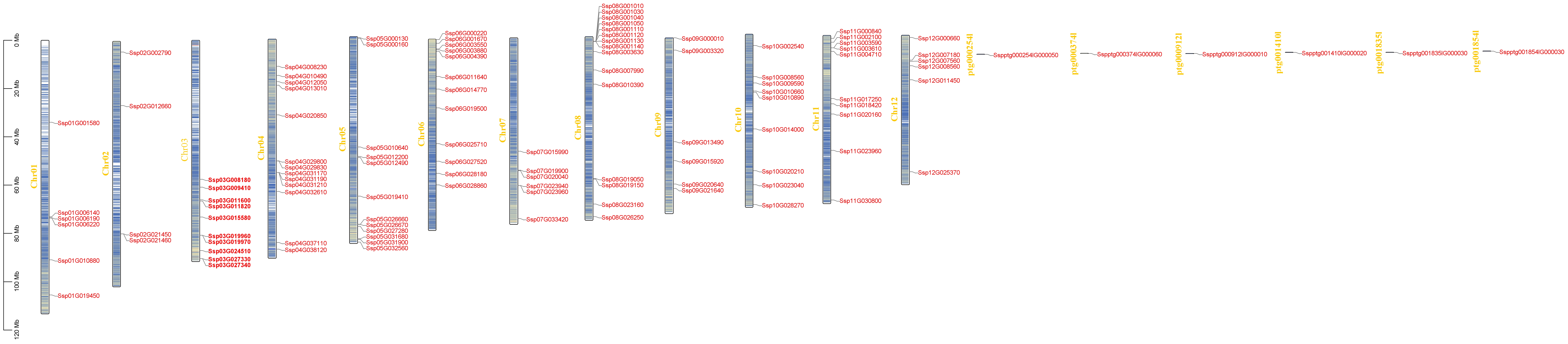

Supplement: Supplementary file 1 [file plants-15-01163-s001.zip › Figures/FigureS1.jpg]

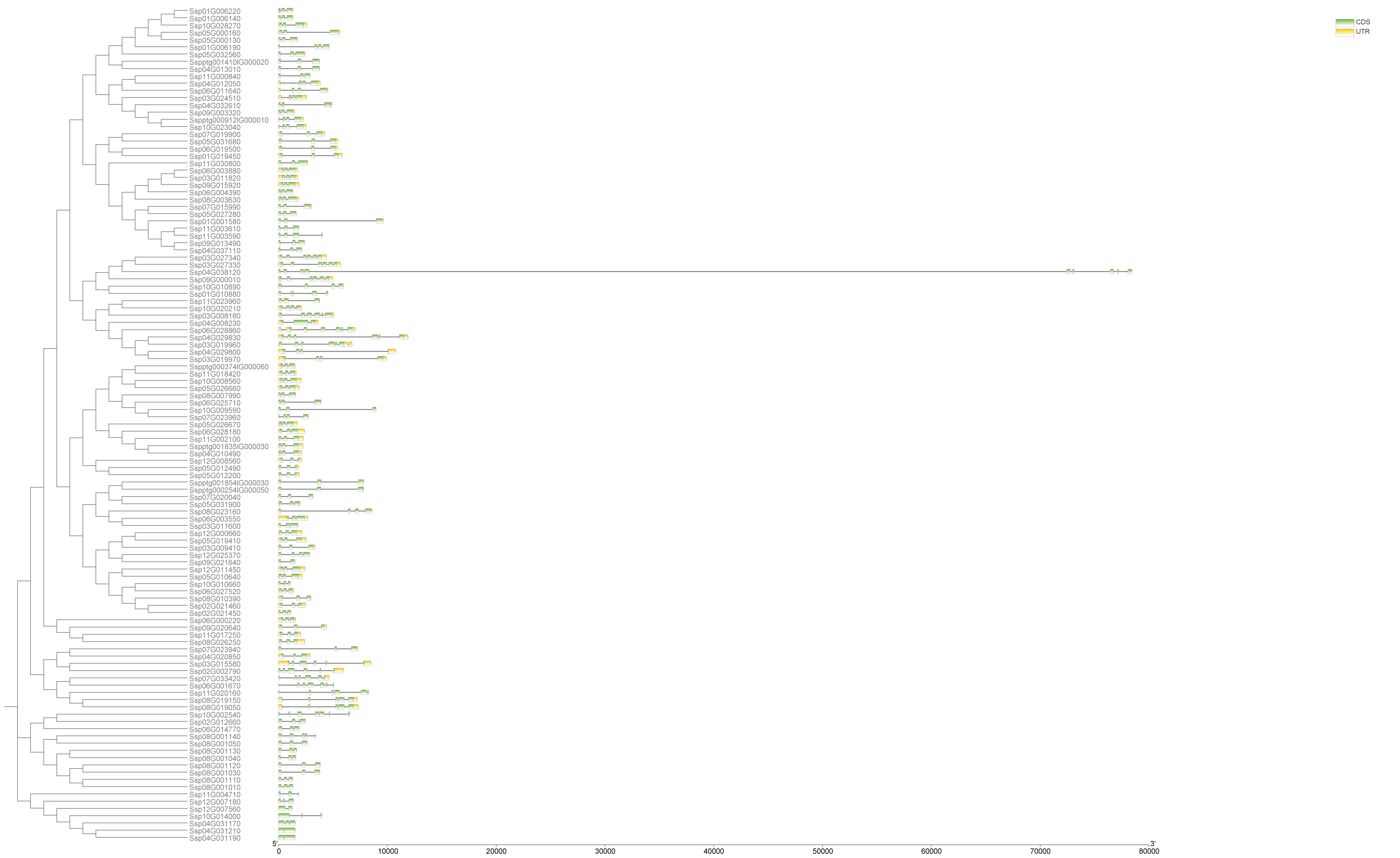

Supplement: Supplementary file 1 [file plants-15-01163-s001.zip › Figures/FigureS2.jpg]

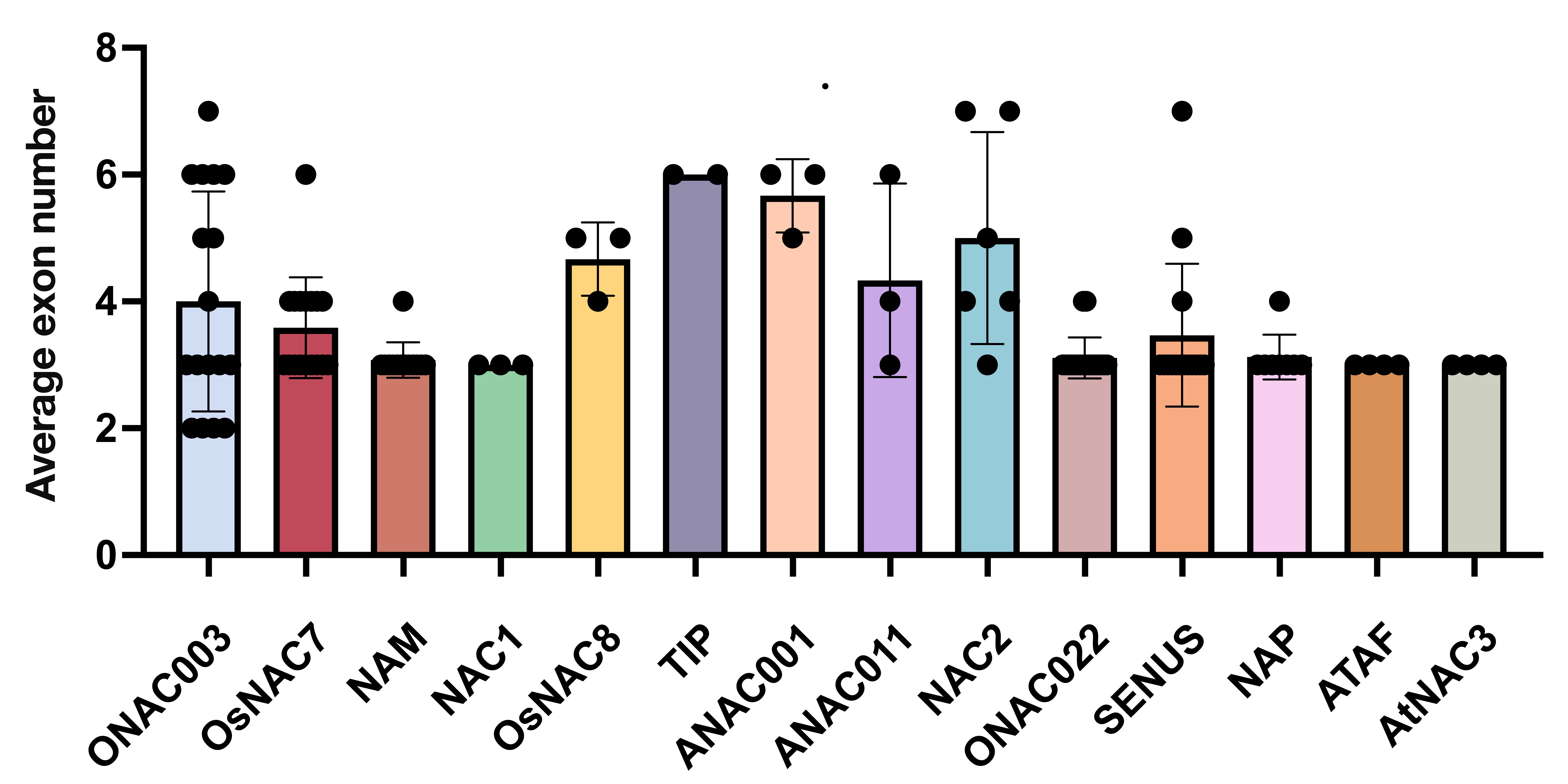

Supplement: Supplementary file 1 [file plants-15-01163-s001.zip › Figures/FigureS3 .jpg]

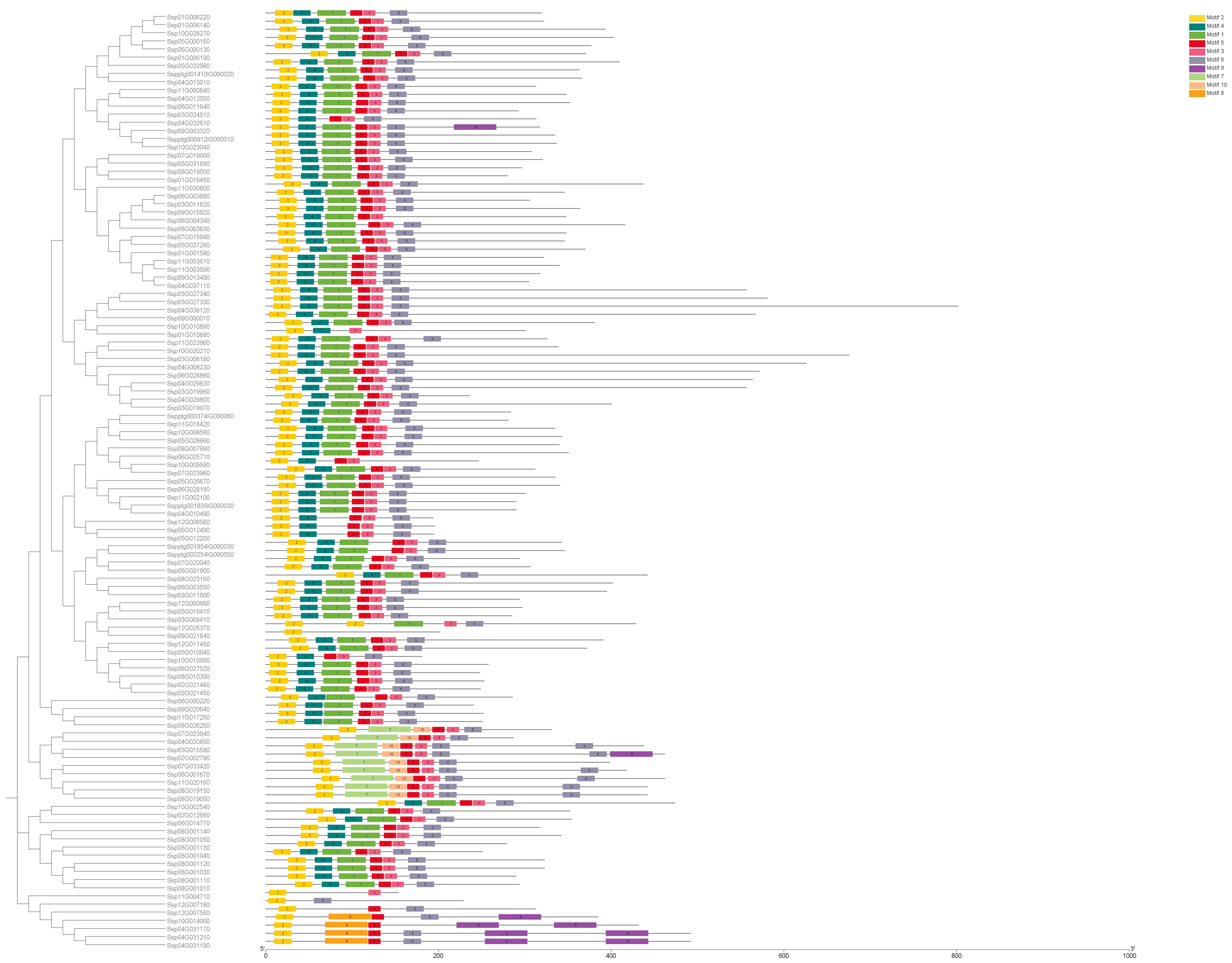

Supplement: Supplementary file 1 [file plants-15-01163-s001.zip › Figures/FigureS4.jpg]

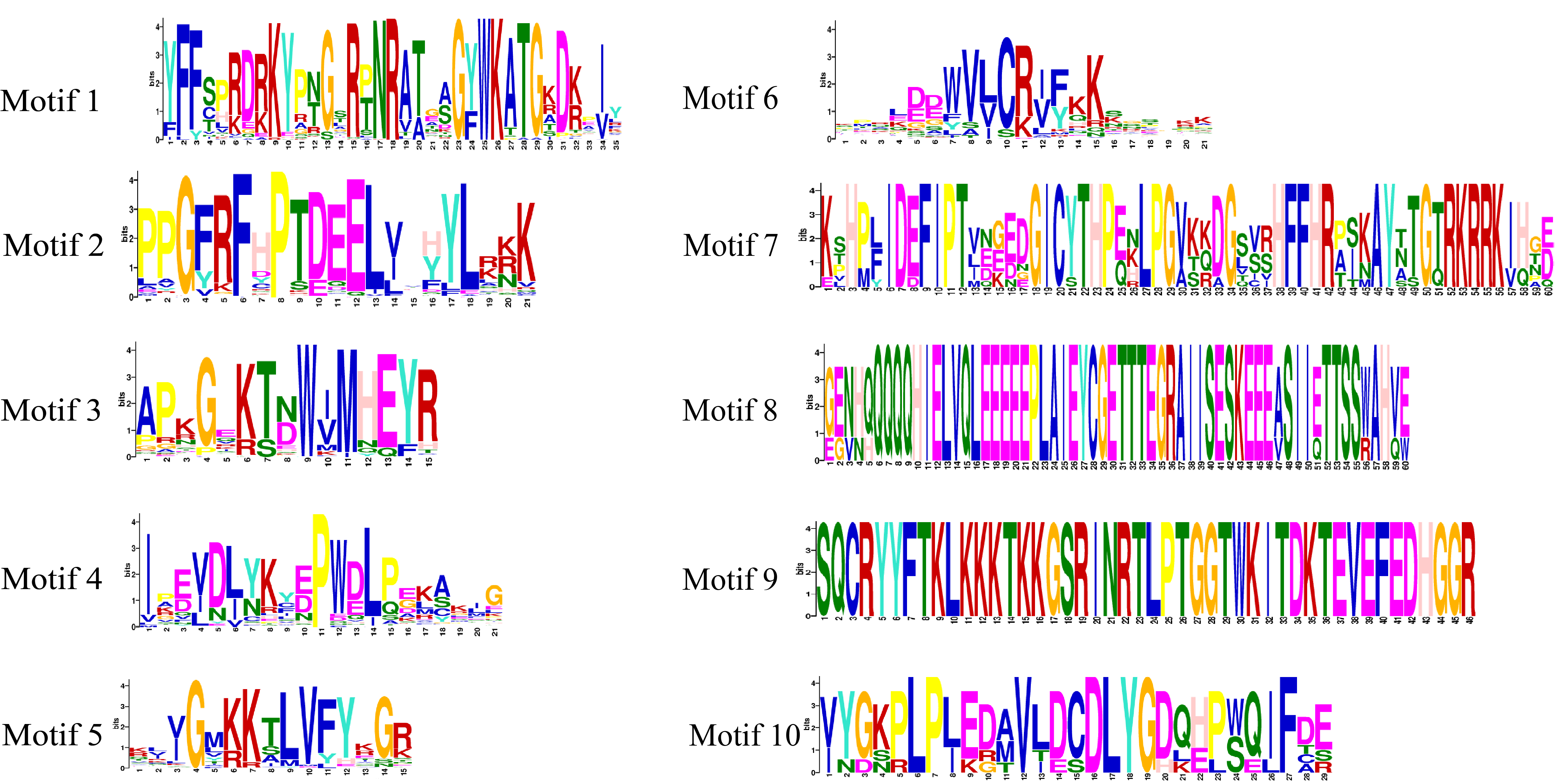

Supplement: Supplementary file 1 [file plants-15-01163-s001.zip › Figures/FigureS5.jpg]

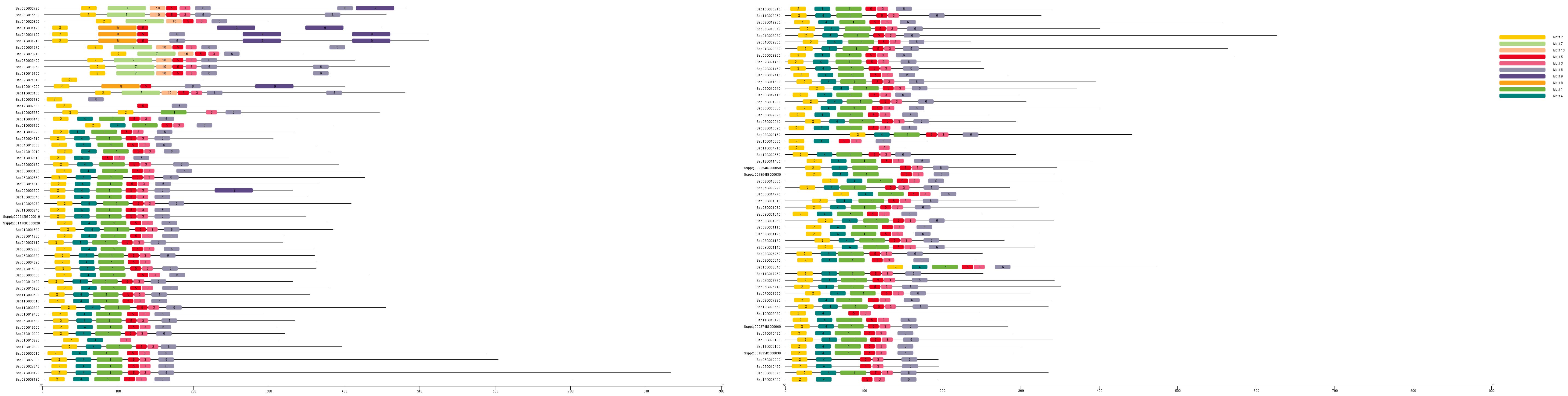

Supplement: Supplementary file 1 [file plants-15-01163-s001.zip › Figures/FigureS6.jpg]

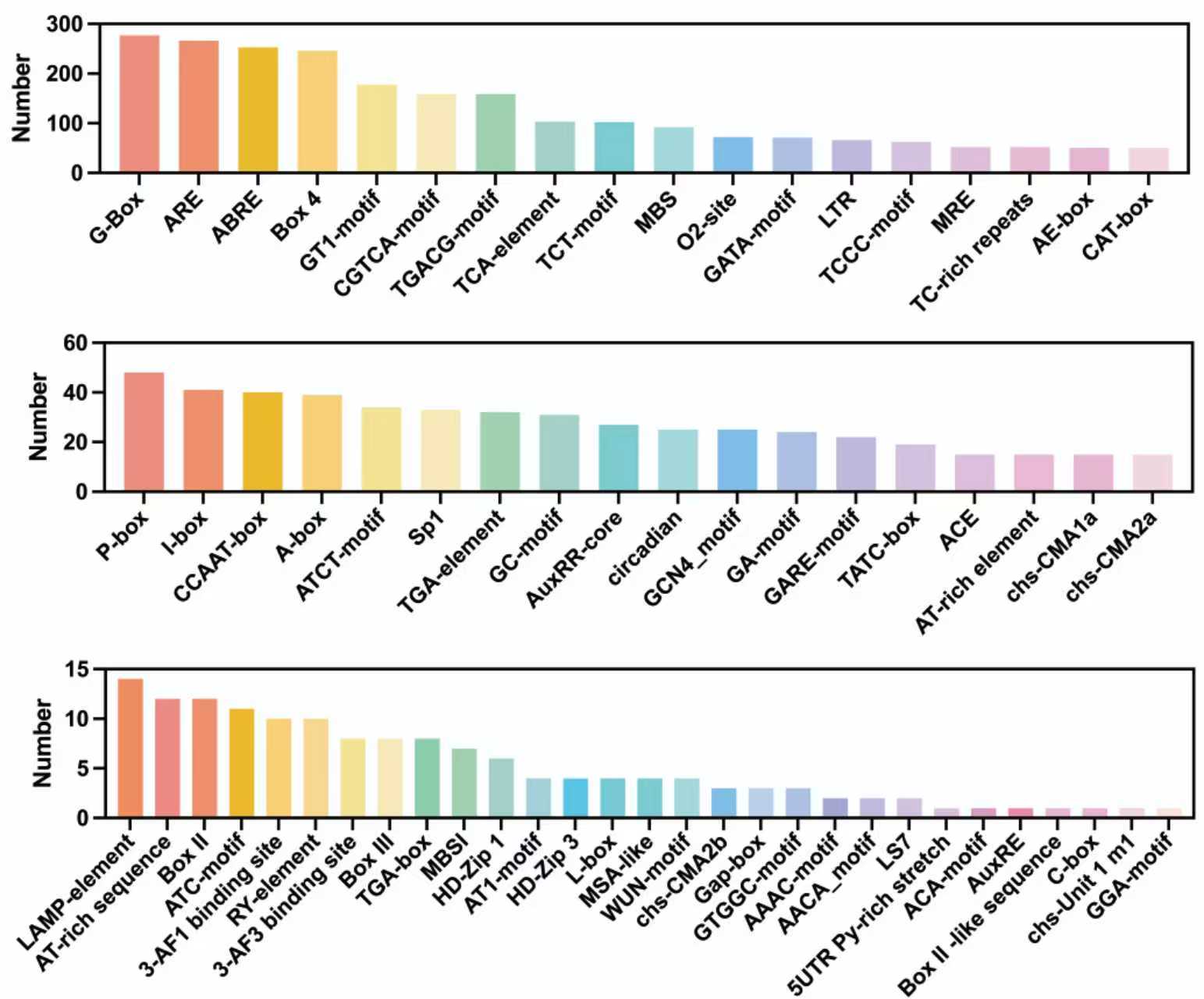

Supplement: Supplementary file 1 [file plants-15-01163-s001.zip › Figures/FigureS7.jpg]

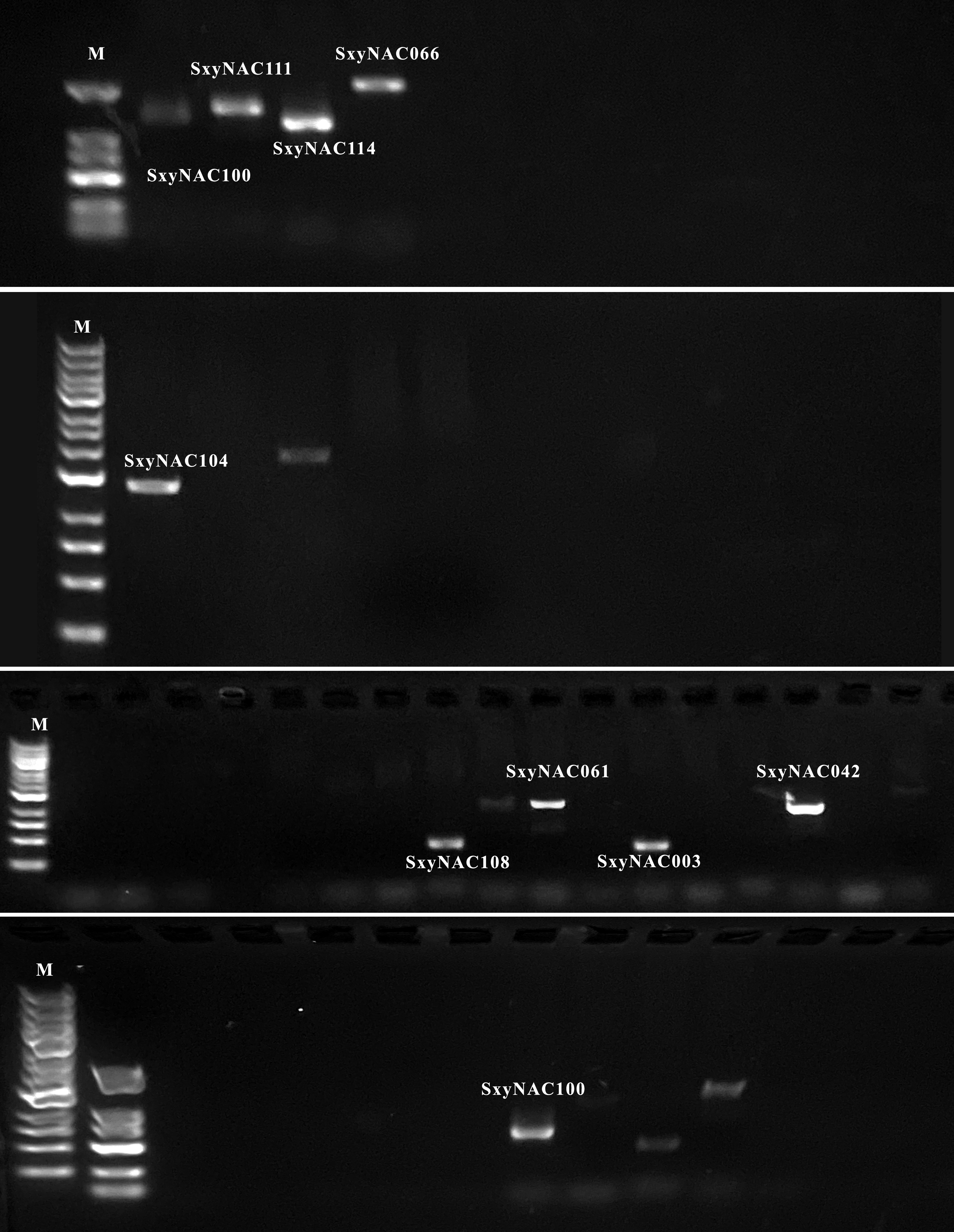

Supplement: Supplementary file 1 [file plants-15-01163-s001.zip › Figures/FigureS9.jpg]
